# *Helicobacter fennelliae* Bacteremia: Three Case Reports and Literature Review

**DOI:** 10.1097/MD.0000000000003556

**Published:** 2016-05-06

**Authors:** Sho Saito, Mika Tsukahara, Kiyofumi Ohkusu, Hanako Kurai

**Affiliations:** From the Division of Infectious Diseases (SS, MT, HK), Shizuoka Cancer Center Hospital, Shimonagakubo, Nagaizumi-cho, Sunto-gun, Shizuoka, Japan; and Department of Microbiology (KO), Tokyo Medical University, Nishishinjuku, Shinjuku-ku, Tokyo, Japan.

## Abstract

*Helicobacter fennelliae* is a gram-negative, spiral bacillus that appears as thin-spread colonies on sheep blood agar and is similar to *Helicobacter cinaedi. H fennelliae* is diagnosed by genetic testing, which is not readily available in all laboratories. Therefore, *H fennelliae* bacteremia has only been reported sporadically, and little is known about its clinical characteristics.

We describe 3 cases of *H fennelliae* bacteremia with gastrointestinal symptoms, including nausea, vomiting, and diarrhea. Isolates could be differentiated from *H cinaedi* by biochemical reaction testing, including nitrate reduction and alkaline phosphatase hydrolysis.

We retrospectively reviewed 24 cases of *H fennelliae* bacteremia reported in the literature. Most of the patients had immunosuppressive backgrounds, including solid tumors, hematological malignancies, and autoimmune diseases. Although gastrointestinal symptoms were common, cellulitis was not often observed in patients with *H fennelliae* bacteremia.

Clinicians should bear in mind that *H fennelliae* may be a differential diagnosis in patients with gastrointestinal manifestations and gram-negative, spiral bacilli. In addition, biochemical reactions, such as nitrate reduction and alkaline phosphatase hydrolysis, are useful in differentiating *H fennelliae* from *H cinaedi.*

## INTRODUCTION

*Helicobacter fennelliae* was first described in 1985 as a new *Campylobacter* species isolated from asymptomatic, homosexual men with enteritis and proctitis.^1^ This organism was subsequently reorganized as a *Helicobacter* species based on 23S rRNA hybridization studies in 1991.^2^*Helicobacter* species are gram-negative, spiral bacteria that are categorized into 2 groups: gastric *Helicobacter* and enterohepatic *Helicobacter.*^3^*H fennelliae* is an enterohepatic *Helicobacter* that causes bacteremia and appears as thin-spread colonies on sheep blood agar. This organism is fastidious and difficult to culture, and its features are similar to *Helicobacter cinaedi*. In addition, *H fennelliae* is diagnosed by genetic testing, such as 16 s rRNA sequencing, which is not readily available in all laboratories. Therefore, *H fennelliae* bacteremia has been reported only sporadically, and little is known about its clinical characteristics.

We report 3 cases of *H fennelliae* bacteremia that could be differentiated from *H cinaedi* by biochemical reaction testing and provide a review of the literature.

## CASE PRESENTATION

### Case 1

A 77-year-old Japanese female with cervical cancer and malignant pleural effusion presented at our hospital. She had a radical hysterectomy for treatment of cervical cancer 1 year prior and had received 3 courses of paclitaxel and nedaplatin. She developed bilateral lower extremity lymphedema 3 days before presentation and low back pain, nausea, and vomiting the day before. Her body temperature was 36.6 °C, heart rate was 105 beats/min, and blood pressure was 93/57 mm Hg. The physical examination revealed tenderness in the upper part of the abdomen, costovertebral angle tenderness, and pitting edema in the lower leg, but was otherwise unremarkable. Blood tests obtained on admission revealed a white blood cell count of 11,730 cells/μL with 97% neutrophils, a C-reactive protein level of 40.5 mg/dL, a blood urea nitrogen level of 58.2 mg/dL, and a creatinine level of 1.39 mg/dL. After 2 sets of blood cultures were obtained, she was treated for dehydration with 1 g of intravenous cefepime, 3 times a day. Five days later, spiral-shaped, gram-negative bacilli, a shape suggestive of *Helicobacter* spp, were isolated from both aerobic blood cultures. Intravenous antibiotic therapy was changed from cefepime to 2 g of ampicillin 4 times a day to treat suspected *H cinaedi* bacteremia. After the patient showed improvement of her general condition, intravenous ampicillin was switched to oral amoxicillin on the 12th day after admission for treatment of enteritis and bacteremia, and antibiotics were given for a total of 18 days. No recurrence was observed during the 18-month follow-up.

### Case 2

A 51-year-old Japanese female with esophageal cancer, liver metastasis, and malignant pleural effusion presented at our hospital. She had received 2 courses of cisplatin and fluorouracil, and radiotherapy. Two days before hospitalization, she had developed anorexia accompanied by nausea and vomiting. Her body temperature was 36.5 °C, heart rate was 98 beats/min, blood pressure was 96/58 mm Hg, and SpO2 was 90%. The physical examination was otherwise unremarkable. Blood tests obtained on admission revealed a white blood cell count of 14,210 cells/μL with 90% neutrophils, a C-reactive protein level of 7.71 mg/dL, a blood urea nitrogen level of 22.0 mg/dL, and a creatinine level of 0.88 mg/dL. Two sets of blood cultures were obtained, and 5 days later, bacteria with a shape suggestive of *Helicobacter* spp were isolated from both blood cultures. The patient was administered 1.5 g of ampicillin/sulbactam intravenously, 4 times a day. However, she died due to an underlying disease 27 days after hospital admission.

### Case 3

A 74-year-old Japanese female with pancreatic cancer and lymph node metastasis, who had received 2 courses of gemcitabine and nanoparticle albumin–bound paclitaxel, was admitted to our hospital due to persistent fever and a positive blood culture. One week before hospitalization, 2 sets of blood cultures were obtained, and spiral-shaped, gram-negative bacilli were isolated from one of the blood cultures after 5 days. Her body temperature on admission was 36.4 °C, heart rate was 63 beats/min, and blood pressure was 112/50 mm Hg. She had a history of diarrhea and pasty stools. Additionally, she noted mild pain in both knees, and pitting edema in the lower leg was observed; however, the physical examination was otherwise unremarkable. Initial laboratory findings included a white blood cell count of 10,800 cells/μL with 76% neutrophils, a C-reactive protein level of 8.24 mg/dL, a blood urea nitrogen level of 16.7 mg/dL, and a creatinine level of 0.77 mg/dL. She was administered 2 g of ampicillin intravenously, 4 times a day. Ampicillin was switched to oral amoxicillin on the 4th day after intravenous treatment, and antibiotics were given for a total of 6 weeks. Her follow-up blood cultures were all negative, and no recurrence had been observed at follow-ups.

**FIGURE 1:**
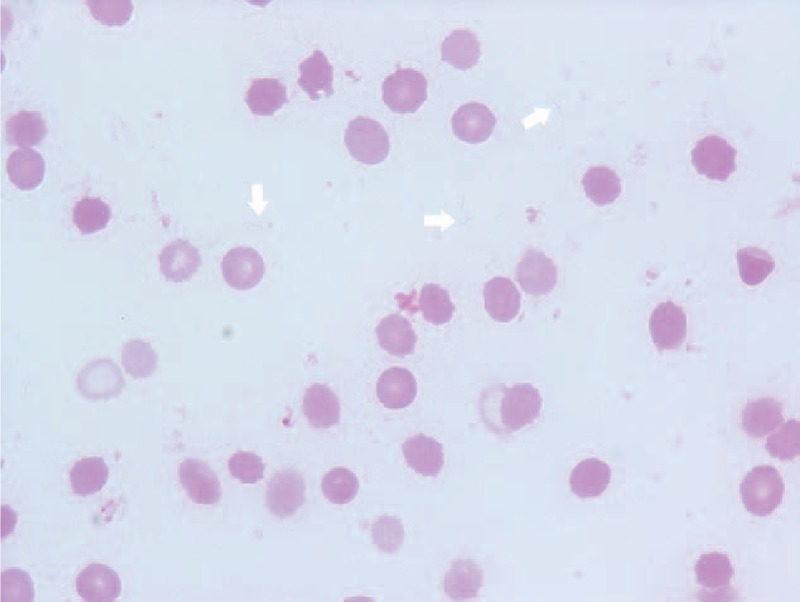
Morphological analysis of *Helicobacter fennelliae* from blood cultures (Gram stain, magnification 1,000×).

**FIGURE 2:**
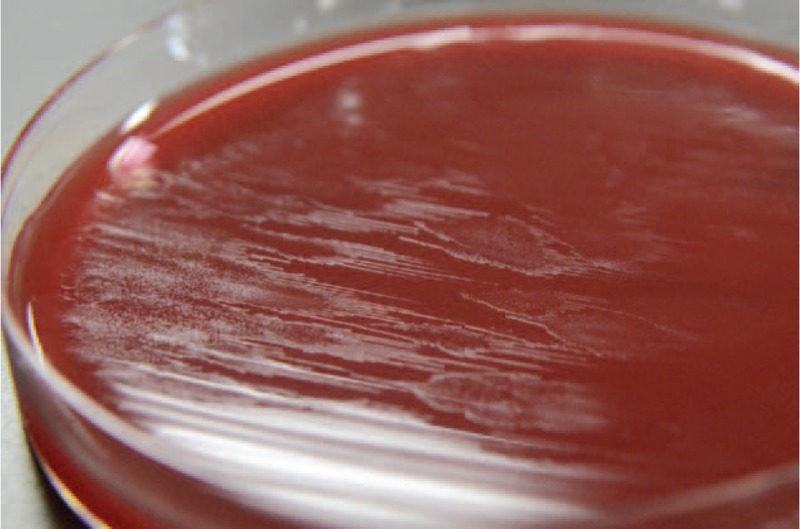
Thin-spread colonies on Trypto soy agar II with sheep blood.

Blood culture samples were processed using the Bactec FX system (Becton, Dickinson and Company, Sparks, MD). Microaerobic cultures were performed with chocolate II agar (Kyokuto Pharmaceutical, Tokyo, Japan) and Trypto soy agar II with sheep blood (Kyokuto Pharmaceutical, Tokyo, Japan) for 6 days at 37 °C in a moist microaerobic atmosphere (5% O_2_, 10% CO_2_, 0% H_2_, 85% N_2_) generated by the TE-HER CAMPYLO INCUBATOR HZC-3 (Hirasawa Works, Tokyo, Japan). *H fennelliae* infection was suspected when blood cultures demonstrated thin-spread colonies and gram-negative spiral bacilli (Figures 1 and 2). The isolates were then identified by DNA sequencing of the 16S rRNA genes for *H fennelliae* and were also tested for nitrate reduction and alkaline phosphatase hydrolysis using the Api campy identification system (bioMerieux Vitek, Tokyo, Japan), which can be performed in general hospitals (Table 1).

**TABLE 1:**
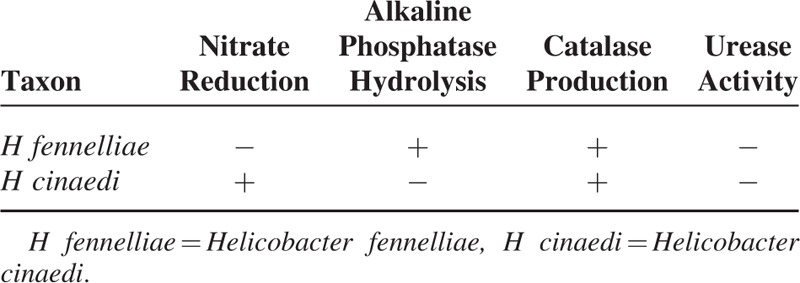
Differential Characteristics of *H fennelliae* and *H cinaedi*

The study protocol was approved by the institutional review board of the Shizuoka Cancer Center Hospital. The patient consent requirement was waived due to the retrospective nature of the study.

## DISCUSSION

According to a growing number of studies and advances in genetic analysis, such as 16S rRNA gene sequencing, the number of reports of *H fennelliae* bacteremia has been steadily growing throughout the last decade. However, few reports have assessed the clinical characteristics or the treatment of patients with *H fennelliae* bacteremia. We describe 3 cases of *H fennelliae* bacteremia that were differentiated from *H cinaedi* by biochemical reaction testing and provide a literature review. To the best of our knowledge, this is the first review of *H fennelliae* bacteremia.

Clinical characteristics of *H fennelliae* bacteremia are summarized in Table 2.^4–17^ A comprehensive literature review of *H fennelliae* bacteremia revealed 24 cases documented between 1993 and 2014. These cases were reported from the United Kingdom, the United States of America, Taiwan, South Africa, and Japan. Skirrow et al first described 2 cases of *H fennelliae* bacteremia in 1993, both of which were in Asian males. Most of the patients had immunosuppressive backgrounds, including solid tumors (4 cases), hematological malignancies (3 cases), diabetes mellitus (1 case), liver diseases (3 cases), kidney diseases (3 cases), autoimmune diseases (3 cases), and organ transplantation (1 case). However, in 1 case, *H fennelliae* bacteremia occurred in a patient who had no known underlying disease. All our cases had solid tumors and had also been receiving chemotherapy as treatment.

**TABLE 2:**
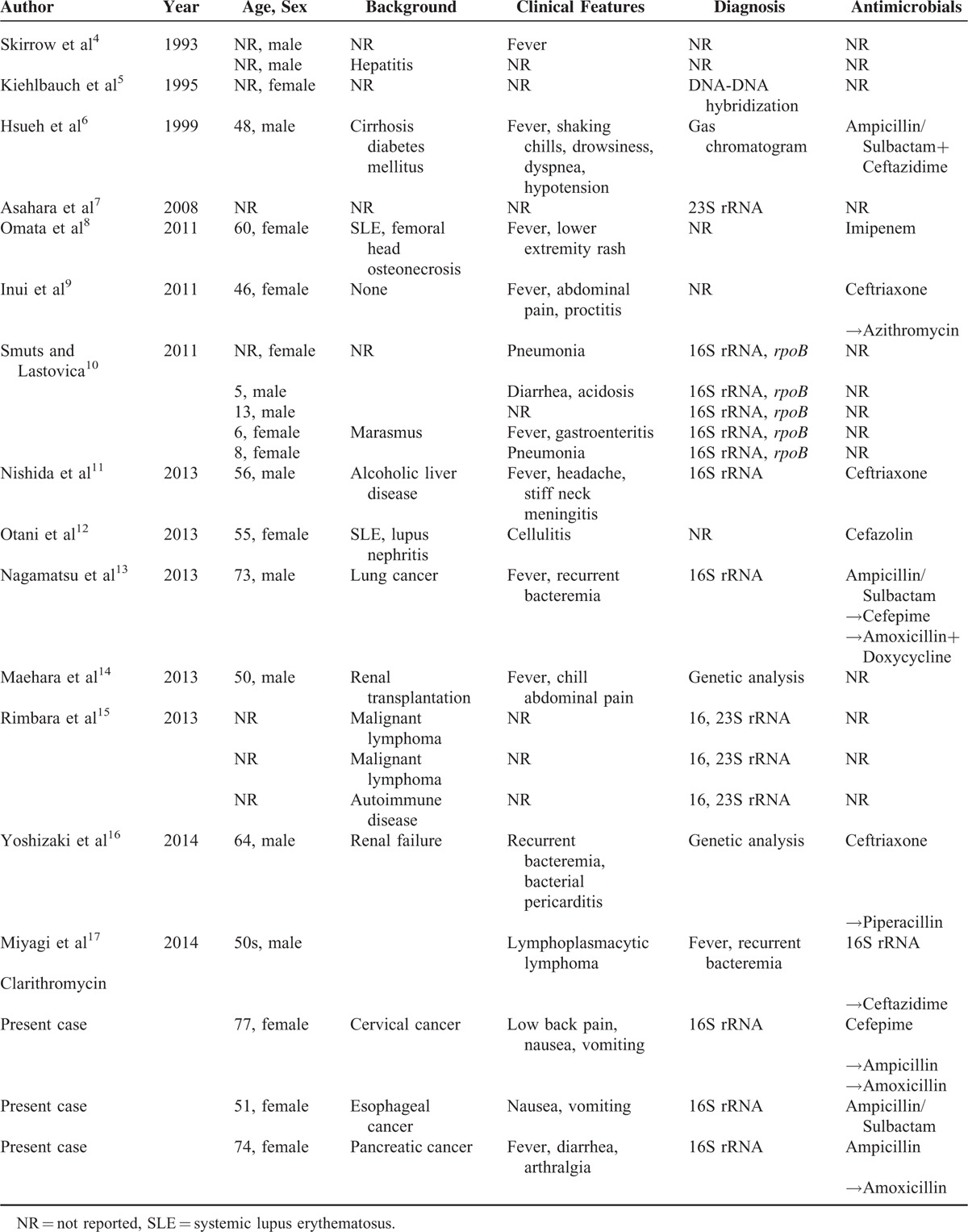
*Helicobacter fennelliae* Bacteremia Case References

Clinical symptoms included gastrointestinal symptoms, such as abdominal pain, diarrhea, nausea, and vomiting (7 cases); cellulitis (1 case), rash (1 case), meningitis (1 case), bacterial pericarditis (1 case), and fever (10 cases). Gastrointestinal symptoms were common; however, cellulitis was not as common in patients with *H fennelliae* bacteremia as it is in those with *H cinaedi* bacteremia.^18^ However, 3 cases of recurrent bacteremia have been identified in previous reports,^13,16,17^ which were similar to those of *H cinaedi*.^18,19^ No deaths have been reported due to *H fennelliae* bacteremia in the current or previous cases.

Detailed pathophysiology of the developing *H fennelliae infection* has not yet been demonstrated. However, acute mucosal inflammation was observed in rectal biopsies from pig-tailed macaque monkeys that developed diarrhea in response to *H fennelliae* infection.^20^ In addition, general and specific mechanisms for innate immune evasion and suppression were established from *Helicobacter* species.^21^ Further research is needed to provide information on the pathophysiology of *H fennelliae infection*.

Currently, there are no recommended guidelines for susceptibility testing or the treatment of diagnosed *H fennelliae* bacteremia. *H cinaedi,* also a gram-negative, spiral bacteria, is well known to be resistant to macrolides and quinolones.^22^ In recent reports, most clinicians have treated *H fennelliae* bacteremia with β-lactam antibiotics as first-line therapy: penicillin in 4 cases, cephalosporin in 6 cases, and carbapenem in 1 case.

Nitrate reduction and alkaline phosphatase hydrolysis reactions were useful in the diagnosis of *H fennelliae*, which has a similar morphology to *H cinaedi. H fennelliae* cultured on an agar plate appears as thin-spread colonies, which are difficult to distinguish from *H cinaedi*. In 1 report, the strain was misidentified as *H cinaedi* because the strains have similar morphologies.^3^ Recently, almost all strains of *H fennelliae* are identified by genetic analysis, such as 16S rRNA gene sequencing; however, such techniques can only be performed in specialized laboratories. *H fennelliae* demonstrate some biochemical differences from other *Helicobacter* species,^23^ such as lacking urease activity and being catalase-positive, nitrate-negative, indoxyl acetate hydrolysis-positive, alkaline phosphatase-positive, and gamma-glutamyl transpeptidase-negative. Tanaka et al reported that nitrate reduction and alkaline phosphatase hydrolysis may be useful in differentiating *Helicobacter* species.^24^ In the present cases, all 3 strains were negative for nitrate reduction, but were alkaline phosphatase-positive, and thus could be differentiated from *H cinaedi*. Therefore, nitrate reduction and alkaline phosphatase hydrolysis tests are useful techniques for diagnosing *H fennelliae* in general laboratories.

In conclusion, we describe 3 cases of *H fennelliae* bacteremia, which had caused gastrointestinal symptoms, and provide a literature review. Although gastrointestinal symptoms were common, cellulitis was not commonly observed in patients with *H fennelliae* bacteremia. Clinicians should bear in mind that *H fennelliae* may be a differential diagnosis in patients with gastrointestinal manifestations and gram-negative, spiral bacilli. In addition, biochemical reaction testing, including nitrate reduction and alkaline phosphatase hydrolysis, is useful in differentiating *H fennelliae* from *H cinaedi*.
